# Address, Delivered before the State Agricultural Society, Members of the Legislature, and of the Medical Society of the State of New-York, at the Capitol in Albany, February, 1848, on the Food of Plants

**Published:** 1848-06

**Authors:** Alexander H. Stevens

**Affiliations:** President of the College of Physicians and Surgeons, and of the Medical Society of the State of New-York, Etc.


*Address, Delivered before the State Agricultural Society, Members of the Legislature, and of the Medical Society of the State of New-York, at the Capitol in Albany, February,* 1848, *on the Food of Plants.* By ALEXANDER H. STEVENS, M. D., President of the College of Physicians and Surgeons, and of the Medical Society of the State of New-York, etc.

Agriculture and Medicine hold to each other a near relationship. Both are concerned with principles of Physiology, Pathology, and Therapeutics. The business of the scientific agriculturist is to study and apply those principles which appertain to the vegetable kingdom, as it is the duty of the physician to investigate and practice upon those which belong to the animal creation. A common department of science embraces both, and often the same principles are involved in the phenomena of vegetable and animal life. It is natural, therefore, for the accomplished physician, when his leisure permits, to turn his attention to agricultural pursuits, and he is peculiarly qualified to excel therein. We should infer from a perusal of the interesting address by Dr. Stevens, that the truth of the foregoing remark is verified in him. The object of the writer is to present in a brief popular manner, some of the fundamental principles of agricultural science—principles pertaining to the Food of Plants. He also advances several original views, which show him to be a practical observer, as well as thinker in this attractive and important province of knowledge. The address must be very acceptable to the intelligent members of the class for which it was intended, and is calculated to do good, by directing attention to the scientific study of Agriculture.

